# Liquid Chromatographic Resolution of Fendiline and Its Analogues on a Chiral Stationary Phase Based on (+)-(18-Crown-6)-2,3,11,12-tetracarboxylic Acid

**DOI:** 10.3390/molecules191221386

**Published:** 2014-12-19

**Authors:** Ga Ram Lee, Myung Ho Hyun

**Affiliations:** Department of Chemistry and Chemistry Institute for Functional Materials, Pusan National University, Busan 690-735, Korea; E-Mail: lgr8969@pusan.ac.kr

**Keywords:** chiral stationary phase, enantiomer separation, liquid chromatography, fendiline

## Abstract

Fendiline, an effective anti-anginal drug for the treatment of coronary heart diseases, and its sixteen analogues were resolved on a CSP based on (+)-(18-crown-6)-2,3,11,12-tetracarboxylic acid. Fendiline was resolved quite well with the separation factor (α) of 1.25 and resolution (R_S_) of 1.55 when a mobile phase consisting of methanol–acetonitrile–trifluoroacetic acid–triethylamine at a ratio of 80/20/0.1/0.5 (v/v/v/v) was used. The comparison of the chromatographic behaviors for the resolution of fendiline and its analogues indicated that the 3,3-diphenylpropyl group bonded to the secondary amino group of fendiline is important in the chiral recognition and the difference in the steric bulkiness between the phenyl group and the methyl group at the chiral center of fendiline is also important in the chiral recognition.

## 1. Introduction

**Figure 1:**
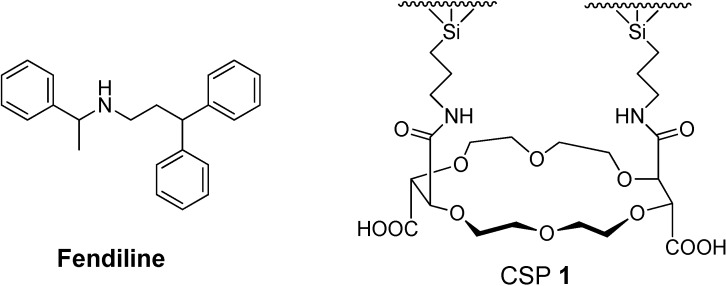
Structures of fendiline and chiral stationary phase (CSP) **1**.

Fendiline (Figure 1) is an effective anti-anginal drug for the treatment of coronary heart diseases. The anti-anginal effects of fendiline have been known to be attributed largely to its dilatory effects on small blood vessels [1]. Fendiline is a chiral drug containing one chiral center. Two enantiomers of chiral drugs have been known often to show different biological activities including pharmacology, toxicology and pharmacokinetics [2]. Two enantiomers of fendiline also have the good chance of showing different biological activities in our body. Actually, the (*R*)-enantiomer of fendiline was found to show a more potent vasodilatory effect than the (*S*)-enantiomer [3]. In this instance, the exact determination of the enantiomeric composition of fendiline is very important. Among various methods, liquid chromatographic chiral stationary phase (CSP) method has been known to be one of the most accurate, convenient and economic means for the determination of enantiomeric composition of chiral drugs [4]. Various liquid chromatographic CSPs have been applied to the determination of enantiomeric composition of chiral compounds [5,6]. For example, CSPs based on polysaccharide derivatives [7], cyclodextrines [8], macrocyclic antibiotics [9,10], cyclofrutanes [11], chiral crown ethers [11,12,13,14] and other small molecules [15] were quite successful for the resolution of racemic compounds. However, liquid chromatographic enantioseparation of fendiline on CSPs is quite rare. Only a CSP based on α_1_-acid glycoprotein (AGP) was used for the enantioseparation of fendiline by liquid chromatography [16].

**Figure 2:**
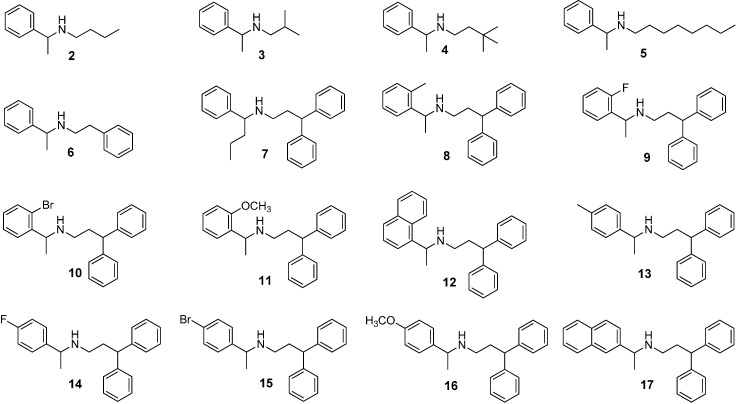
Structures of fendiline analogues (**2**–**17**).

CSPs based on chiral crown ethers were very successful in the determination of enantiomeric composition of chiral compounds containing a primary amino group [12,17,18,19]. While CSPs based on chiral crown ethers have been known to be effective for the resolution of racemic compounds containing a primary amino group through the enantioselective complexation of the primary ammonium group (R-NH_3_^+^) of analytes inside the cavity of the crown ether ring of the stationary phase [12,17,18,19], interestingly CSP **1** (Figure 1) based on (+)-(18-crown-6)-2,3,11,12-tetracarboxylic acid was found to be also quite successful in the resolution of racemic compounds containing a secondary amino group such as β-blockers [20], flecainide analogues [21], isoquinolines [22] and rasagiline analogues [23]. Fendiline also contains a secondary amino group and, in this instance, it is expected to be resolved on CSP **1**. In this study, we wish to report the resolution of fendiline and its analogues (**2**–**17**) shown in Figure 2 on CSP **1**.

## 2. Results and Discussion

The structural characteristics required for the resolution of fendiline on CSP **1** might be elucidated by comparing the chromatographic resolution behaviors for the resolution of fendiline and its analogues. Consequently, various types of fendiline analogues shown in Figure 2 were prepared. In order to see the importance of the 3,3-diphenylpropyl group of fendiline, analogues (**2**–**6**) containing simple straight chain alkyl group, sterically bulky alkyl group or phenylalkyl group at the nitrogen of the secondary amino group were prepared. Analogue (**7**) containing a propyl group instead of the methyl group at the chiral center of fendiline was also prepared to see the effect of the methyl group on the chiral resolution. Analogues (**8**–**12**) containing ortho-substituted phenyl or 1-naphthyl group and analogues (**13**–**17**) containing para-substituted phenyl or 2-naphthyl group instead of the simple phenyl group were prepared for the purpose of elucidating the role of the phenyl group at the chiral center of fendiline in the chiral recognition.

For the resolution of secondary amino compounds on CSP **1**, mobile phase composition has been known to be very important. For the resolution of secondary amino alcohols related to β-blockers on CSP **1**, a mixture of ethanol–acetonitrile–trifluoroacetic acid–triethylamine (20/80/0.1/0.5, v/v/v/v) was successfully used as a mobile phase [20]. However, for the resolution of flecainide and its analogues on CSP **1**, a different mobile phase consisting of methanol–acetonitrile–trifluoroacetic acid–triethylamine (80/20/0.1/0.3, v/v/v/v) was most widely applied [21]. For the resolution of rasagiline and analogues on CSP **1**, another mixture of ethanol–acetonitrile–acetic acid–triethylamine (80/20/0.2/0.3, v/v/v/v) was most widely applied as a mobile phase [23]. From these results, a certain mixture of ethanol or methanol in acetonitrile containing a small amount of acidic (acetic or trifluoroacetic acid) and basic (triethylamine) modifier is expected to be used as a mobile phase for the resolution of fendiline and its analogues on CSP **1**. To find out the most widely applicable mobile phase condition for the resolution of fendiline and its analogues, we selected five analytes including fendiline and its analogues **8**, **12**, **13** and **17**. Analytes **8** and **12** were selected to represent analogues (**8**–**12**) containing ortho-substituted phenyl or 1-naphthyl group and analytes **13** and **17** were selected to represent analogues (**13**–**17**) containing para-substituted phenyl or 2-naphthyl group instead of the simple phenyl group at the chiral center of fendiline. The selected five analytes were resolved on CSP **1** with the variation of the mobile phase composition. The resolutions of the selected five analytes on CSP **1** with the variation of the type and content of alcohol in acetonitrile at the constant ratio of trifluoroacetic acid-triethylamine (0.1/0.5, v/v) are summarized in Table 1. The retention factors (*k*_1_) for the resolution of selected five analytes were found to increase as the content of methanol in acetonitrile was increased from 50% to 80%. In addition, the retention factors (*k*_1_) for the resolution of selected five analytes were also found to increase as the type of alcohol in acetonitrile was changed from methanol to ethanol and then to 2-propanol. By increasing the content of methanol in acetonitrile, the mobile phase polarity seems to decrease. By changing the type of alcohol in acetonitrile from methanol to ethanol and then to 2-propanol, the mobile phase polarity is also expected to decrease continuously. When the mobile phase polarity is decreased, the interaction between the mobile phase and analytes is diminished and, consequently, the retention factors (*k*_1_) increase. However, the separation factors (α) and resolutions (R_S_) for the resolution of selected five analytes on CSP **1** did not show any significant trends. For fendiline, **12** and **13**, 80% methanol in acetonitrile was found to show the best resolution results especially in terms of the resolutions (R_S_). However, for the resolution of **8** and **17**, the best resolution results in terms of both the separation factors (α) and resolutions (R_S_) were obtained when 80% ethanol in acetonitrile or 50% methanol in acetonitrile, respectively, was used.

As an effort to find out the optimum content of acidic and/or basic modifier in the mobile phase, the five analytes were resolved on CSP **1** with the variation of the ratio of trifluoroacetic acid and triethylamine in 80% methanol in acetonitrile and the resolution results are summarized in Table 2. As the content of trifluoroacetic acid is increased from 0.05% to 0.1% and then 0.2% (entry a, b and e) with a constant content of triethylamine (0.5%), the retention factors (*k*_1_) increased continuously. However, the retention factors (*k*_1_) increased continuously as the content of triethylamine is decreased from 0.75% to 0.5% and then to 0.25% (entry d, b and c) with a constant content of trifluoroacetic acid (0.1%). The retention factors (*k*_1_) also increased as the content of triethylamine is decreased from 0.75% and then to 0.50% (entry f and e) with a constant content of trifluoroacetic acid (0.20%). In general, the retention factors (*k*_1_) increase as the content of trifluoroacetic acid is increased and as the content of triethylamine is decreased. As the content of trifluoroacetic acid is increased or as the content of triethylamine is decreased in the mobile phase, the protonation state of the analyte amino group is expected to increase. In this instance, the interaction between the protonated amino group of analytes and the CSP increases and, consequently, the retention factors are expected to increase as the content of trifluoroacetic acid is increased or as the content of triethylamine is decreased in the mobile phase.

While the retention factors (*k*_1_) show some trends with the variation of the ratio of trifluoroacetic acid and triethylamine in 80% methanol in acetonitrile as shown in Table 2, the separation factors (α) were found not to show any specific trend. The separation factors (α) were found to vary only in small range with the variation of the ratio of trifluoroacetic acid and triethylamine in the mobile phase. The resolutions (R_S_) were also found not to show any specific trend. When the ratio of trifluoroacetic acid and triethylamine was 0.10/0.25 (v/v, entry c), the retention factors (*k*_1_) were highest, but the separation factors (α) and resolutions (R_S_) were worst. For the resolution of fendiline, the baseline resolution (R_S_ is >1.5) was obtained only with the use of a mobile phase consisting of methanol–acetonitrile–trifluoroacetic acid–triethylamine at a ratio of 80/20/0.1/0.5 (v/v/v/v) (entry b in Table 2).

**Table 1:** Resolution of racemic fendiline and its analogues (**8**, **12**, **13** and **17**) on CSP **1** with the variation of the content of methanol (MeOH), ethanol (EtOH) or 2-propanol (PrOH) in acetonitrile (ACN) as a mobile phase containing trifluoroacetic acid (TFA)-triethylamine (TEA) of the constant ratio [MeOH or EtOH or iPrOH-ACN-TFA-TEA, x/(100 − x)/0.1/0.5, v/v/v/v]. Flow rate: 0.5 mL/min. Detection: 254 nm UV. Column temperature: 20 °C. *k*_1_: Retention factor of the first eluted enantiomer. α: Separation factor. R_S_: Resolution.

Alcohol Content (x)	Fendiline			8			12			13			17		
	*k*_1_	α	R_S_	*k*_1_	α	R_S_	*k*_1_	α	R_S_	*k*_1_	α	R_S_	*k*_1_	α	R_S_
50% MeOH	1.21	1.22	0.92	1.13	1.24	0.97	1.28	1.35	1.48	1.13	1.24	0.97	1.28	1.35	1.48
80% MeOH	1.91	1.25	1.55	2.36	1.25	1.31	2.08	1.28	1.15	2.36	1.25	1.31	2.08	1.28	1.15
80% EtOH	3.20	1.27	1.09	4.06	1.27	1.13	3.12	1.31	0.89	4.06	1.27	1.13	3.12	1.31	0.89
80% PrOH	4.49	1.23	0.76	5.64	1.23	0.59	5.25	1.26	1.01	5.64	1.23	0.59	5.25	1.26	1.01

**Table 2:** Resolution of racemic fendiline and its analogues (**8**, **12**, **13** and **17**) on CSP **1** with the variation of the ratio of trifluoroacetic acetic acid (TFA)-triethylamine (TEA) in 80% methanol in acetonitrile as a mobile phase (MeOH-ACN-TFA-TEA, 80/20/x/y, v/v/v/v). Flow rate: 0.5 mL/min. Detection: 254 nm UV. Column temperature: 20 °C. *k*_1_: Retention factor of the first eluted enantiomer. α: Separation factor. R_S_: Resolution.

Entry	TFA/TEA Ratio (v/v)	Fendiline			8			12			13			17		
		*k*_1_	α	R_S_	*k*_1_	α	R_S_	*k*_1_	α	R_S_	*k*_1_	α	R_S_	*k*_1_	α	R_S_
a	0.05/0.5	1.57	1.27	1.19	1.81	1.41	1.60	1.61	1.50	2.56	2.08	1.25	1.16	1.88	1.29	1.52
b	0.1/0.5	1.91	1.25	1.55	1.98	1.39	1.24	1.82	1.54	2.50	2.36	1.25	1.31	2.08	1.28	1.15
c	0.1/0.25	4.30	1.10	0.48	4.37	1.23	0.88	4.40	1.33	1.73	5.25	1.13	0.83	4.75	1.15	0.74
d	0.1/0.75	1.16	1.21	0.78	1.39	1.37	1.38	1.23	1.47	2.11	1.69	1.22	1.19	1.46	1.25	1.42
e	0.2/0.5	2.55	1.25	1.20	3.03	1.40	1.41	2.87	1.48	1.92	3.15	1.24	1.09	3.15	1.28	1.33
f	0.2/0.75	1.49	1.19	0.84	1.49	1.32	1.01	1.47	1.45	1.85	2.05	1.21	1.47	1.66	1.23	0.98

Resolution of analytes **12** and **13** was also quite good with the use of a mobile phase consisting of methanol–acetonitrile–trifluoroacetic acid–triethylamine at a ratio of 80/20/0.1/0.5 (v/v/v/v). However, a mobile phase consisting of methanol–acetonitrile–trifluoroacetic acid–triethylamine at a ratio of 80/20/0.05/0.5 (v/v/v/v) (entry a in Table 2) was found to be the best mobile phase condition for the resolution of analytes **8** and **17** in terms of both the separation factors and resolutions. As an acidic modifier, acetic acid was also tested instead of trifluoroacetic acid. However, the mobile phase containing acetic acid was found to be inferior to that containing trifluoroacetic acid. For example, fendiline was resolved with the separation factor (α) of 1.25 and resolution (R_S_) of 1.55 when a mobile phase consisting of methanol–acetonitrile–trifluoroacetic acid–triethylamine at a ratio of 80/20/0.1/0.5 (v/v/v/v) was used, but it was resolved with the separation factor (α) of 1.24 and resolution (R_S_) of 1.13 when a mobile phase consisting of methanol–acetonitrile–acetic acid–triethylamine at a ratio of 80/20/0.1/0.5 (v/v/v/v) was used.

Column temperature is also an important factor for the resolution of racemic primary and secondary amino compounds on CSP **1** [12]. For the resolution of racemic compounds containing a primary amino group on CSP **1**, the separation factors (α) have been reported to increase always as the column temperature was decreased as usual [12]. However, for the resolution of β-blockers containing a secondary amino group on CSP **1**, the separation factors (α) were found, very surprisingly and unusually, to increase as the column temperature was increased [20]. Based on the van’t Hoff plots, both ΔΔH and ΔΔS values for the resolution of β-blockers on CSP **1** were calculated to be positive and, consequently, the negative ΔΔG values corresponding to the separation factor (α) of greater than 1.00 were concluded to be entirely dependent on the ΔΔS values, indicating that the resolution of β-blockers on CSP **1** is entropy controlled [20]. Fendiline is a secondary amino compound. In this instance, the resolution of fendiline on CSP **1** is expected to follow the unusual temperature effect on the chiral recognition. However, when the column temperature was changed from 30 to 20 and then to 10 °C for the resolution of fendiline on CSP **1**, the separation factor (α) increased continuously from 1.21 to 1.25 and then to 1.32. These results indicate that the resolution of fendiline on CSP **1** follows the usual temperature effect on the chiral recognition.

**Figure 3:**
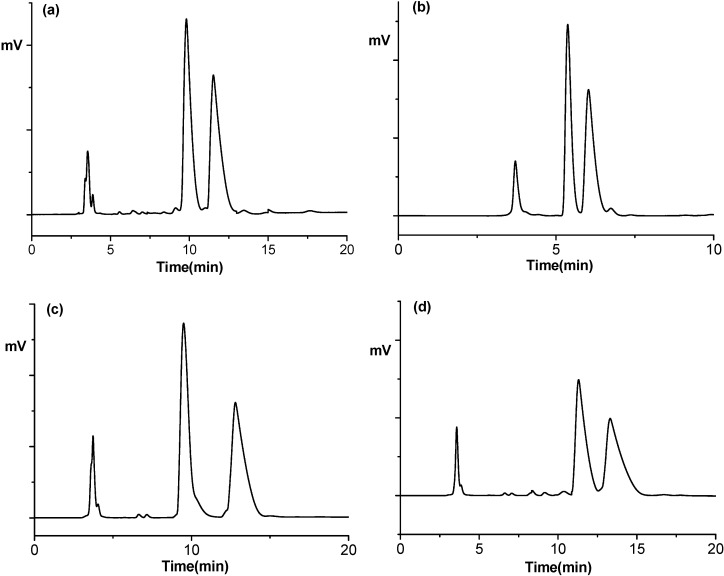
Representative chromatograms for the resolution of (**a**) fendiline and its analogues; (**b**) **10**; (**c**) **12**; and (**d**) **13** with the use of a mobile phase consisting of methanol–acetonitrile–trifluoroacetic acid–triethylamine at a ratio of 80/20/0/1/0.5 (v/v/v/v). Flow rate: 0.5 mL/min. Detection: 254 nm UV. Temperature, 20 °C.

In order to compare the chromatographic behavior for the resolution of fendiline with those for the resolution of its analogues (**2**–**17**) on CSP **1**, all analytes were resolved with the use of a mobile phase consisting of methanol–acetonitrile–trifluoroacetic acid–triethylamine at a ratio of 80/20/0.1/0.5 (v/v/v/v), the best mobile phase condition for the resolution of fendiline, and the resolution results are summarized in Table 3 and the representative chromatograms are illustrated in Figure 3. The elution orders for fendiline and its analogues **4**, **6**, **12** and **17** shown in Table 3 were determined by injecting configurationally known optically active samples, which were prepared from the commercially available optically active 1-phenylethylamine, 1-(α-naphthyl)ethylamine or 1-(β-naphthyl)ethylamine. For other analytes, the elution orders were not determined because the elution orders were not clear (for analogues **3** and **5**) or optically active samples were not able to be prepared due to the lack of optically active starting materials (for analogues **7**–**11** and **13**–**16**).

**Figure 4:**
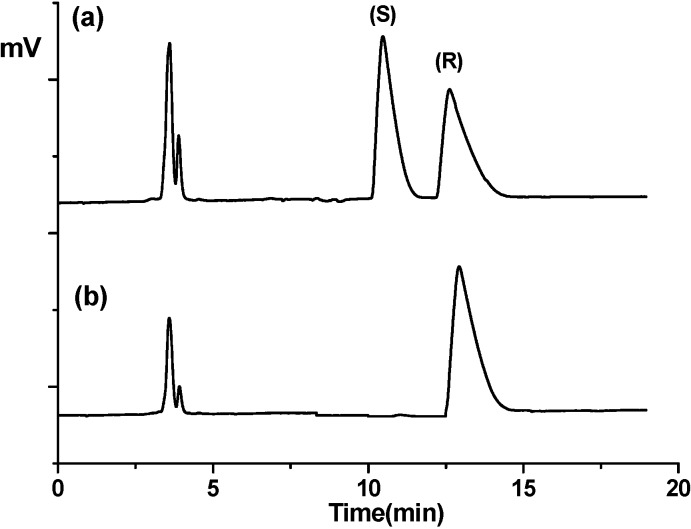
Comparison of the chromatograms for the resolution of (**a**) racemic fendiline and (**b**) (*R*)-fendiline on CSP **1** with the use of a mobile phase consisting of methanol–acetonitrile–trifluoroacetic acid–triethylamine at a ratio of 80/20/0.1/0.5 (v/v/v/v). Flow rate: 0.5 mL/min. Detection: 254 nm UV. Temperature, 20 °C.

Interestingly, the baseline resolution of fendiline makes CSP **1** useful for the determination of the enantiomeric purity or enantiomeric composition of biologically more active (*R*)-fendiline. The comparison of the chromatograms shown in Figure 4 for the resolution of racemic fendiline and optically active (*R*)-fendiline prepared from (*R*)-phenylethylamine of more than 99% ee demonstrates the usefulness of CSP **1** for the determination of enantiomeric purity of (*R*)-fendiline. From Figure 4, the enantiomeric purity of the (*R*)-fendiline is concluded to be more than 99% ee because the peak corresponding to the (*S*)-enantiomer is not shown in the chromatogram obtained from the sample of (*R*)-fendiline.

**Table 3:** Resolution of racemic fendiline and its analogues (**2**–**17**) on CSP **1** with the use of a mixture of methanol–acetonitrile–trifluoroacetic acid–triethylamine (MeOH–ACN–TFA–TEA, 80/20/0.1/0.5, v/v/v/v) as a mobile phase. Flow rate: 0.5 mL/min. Detection: 254 nm UV. Temperature, 20 °C; *k*_1_, retention factor of the first eluted enantiomer; *k*_2_, retention factor of the second eluted enantiomer. Absolute configurations of the first and second eluted enantiomers are indicated in the parenthesis. α, separation factor; R_S_, resolution.

Analytes	*k*_1_	*k*_2_	α	R_S_
**fendiline**	1.91(S)	2.42(R)	1.25	1.55
**2**	1.56	1.76	1.13	0.42
**3**	0.95	1.03	1.08	0.48
**4**	1.61(S)	1.85(R)	1.15	0.74
**5**	1.33	1.50	1.13	0.58
**6**	0.92(S)	1.04(R)	1.12	0.74
**7**	1.64	2.14	1.31	0.87
**8**	1.98	2.76	1.39	1.24
**9**	0.69	0.89	1.28	1.27
**10**	0.59	0.79	1.33	1.42
**11**	2.39	3.02	1.26	1.23
**12**	1.82(S)	2.80(R)	1.54	2.50
**13**	2.36	2.95	1.25	1.31
**14**	1.57	1.92	1.22	1.12
**15**	1.61	1.97	1.22	0.79
**16**	3.10	3.77	1.22	0.97
**17**	2.08(S)	2.65(R)	1.28	1.15

When the 3,3-diphenylpropyl group at the secondary nitrogen atom of fendiline was replaced with simple *n*-alkyl, sterically bulky alkyl or 2-phenylalkyl group (analytes **2**–**6**), both of the separation factors and resolutions were decreased quite much. Consequently, the 3,3-diphenylpropyl group bonded to the secondary amino group of fendiline is expected to play an important role in the chiral recognition. When the methyl group at the chiral center of fendiline was changed as a longer alkyl group (analyte **7**), both of the separation factors and resolutions were also decreased quite much, indicating the importance of the methyl group at the chiral center in the chiral recognition. By changing the methyl group as a longer alkyl group at the chiral center of fendiline, the size difference between the two groups at the chiral center of analyte is expected to be diminished and, consequently, the discrimination of the two enantiomers might become less effective. When the phenyl group at the chiral center of fendiline was replaced with ortho-substituted phenyl group (analytes **8**–**11**) or 1-naphthyl group (analyte **12**), the separation factors increased, but the resolutions decreased compared with that for fendiline except for analyte **12**. However, the phenyl group at the chiral center of fendiline was replaced with para-substituted phenyl group (analytes **13**–**16**) or 2-naphthyl group (analyte **17**), the separation factors were not changed much, but the resolutions decreased in every case compared with that of fendiline. By replacing the phenyl group at the chiral center of fendiline with an ortho-substituted phenyl group, the steric bulkiness of the substituent experienced at the chiral center of analytes is expected to increase and, consequently, the size difference between the two groups at the chiral center of analyte is expected to increase and, consequently, the discrimination of the two enantiomers might become more effective. The steric bulkiness experienced at the chiral center of analytes with the ortho-substituted phenyl group seems to be greater than that experienced with the para-substituted phenyl group. In this instance, an analyte containing an ortho-substituted phenyl group should show greater chiral recognition than the corresponding analyte containing a para-substituted phenyl group. In particular, analyte **12** containing 1-naphthyl group at the chiral center was resolved much better than fendiline. The 1-naphthyl group at the chiral center of analyte **12** seems to be much bulkier group than the ortho- or para-substituted phenyl group or 2-naphthyl group and, consequently, analyte **12** is expected to be resolved best. However, the exact chiral recognition mechanism is not clear yet.

## 3. Experimental Section

### 3.1. Chromatography

Liquid chromatographic results for the resolution of fendiline and its analogues were obtained with an HPLC system consisting of a Rheodyne model 7725i injector (Rohnert Park, CA, USA) with a 20 μL sample loop, a Waters model 515 HPLC pump (Milford, MA, USA), a Waters 484 Tunable Absorbance detector and a YoungLin Autochro data module (Software: YoungLin Autochro-WIN 2.0 plus). The chiral column temperature was maintained at 20 °C by using a JEIO TECH VTRC-620 cooling circulator (Daejeon, Korea). Chiral column packed with end-capped CSP **1** [Chirosil RCA(+), 150 mm × 4.6 mm I.D.] was available from RS tech (Daejeon, Korea). The number of theoretical plates (N) of the chiral column calculated from the retention time and the peak width at half height for the first eluted enantiomer of fendiline was 2537. Injection samples were prepared by dissolving each analyte in chloroform at a concentration of 1.0 mg/mL. The injection volume was usually 1.0 μL.

### 3.2. Preparation of Fendiline and Its Analogues

**Figure 5:**
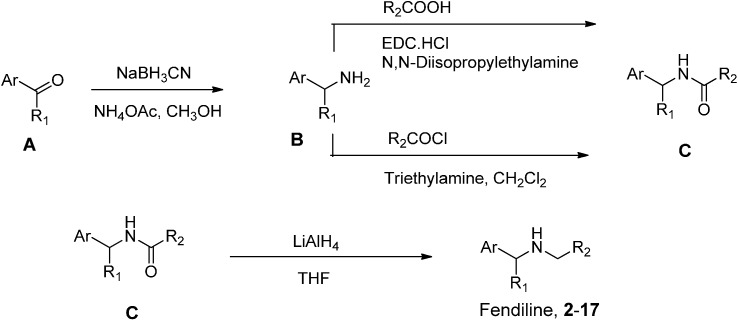
Scheme for the preparation of fendiline and its analogues (**2**–**17**).

Fendiline and its analogues were prepared according to the scheme shown in Figure 5. Starting materials for the preparation of fendiline and its analogues **2**–**6**, **12** and **17** including 1-phenylethylamine (Ar = phenyl, R_1_ = CH_3_, compound B in Figure 5), 1-(α-naphthyl)ethylamine (Ar = α-naphthyl, R_1_ = CH_3_, compound B in Figure 5) and 1-(β-naphthyl)ethylamine (Ar = β-naphthyl, R_1_ = CH_3_, compound B in Figure 5) were commercially available from Sigma-Aldrich (St. Louis, MO, USA). Starting materials for the preparation of fendiline analogues **7**–**11** and **13**–**16** such as 1-(substituted phenyl)ethylamines and 1-phenylbutylamine were prepared through the reductive amination of appropriate aryl alkyl ketones (compound A in Figure 5) by treating aryl alkyl ketones with sodium cyanoborohydride (NaCNBH_3_) and ammonium acetate (NH_4_OAc) in methanol. Compound B then was treated with carboxylic acid in the presence of 1-ethyl-3-(3-dimethylaminopropyl)carbodiimide hydrochloride (EDC·HCl, carboxylic acid group activating reagent) and *N*,*N*-diisopropylethylamine (DIEA) in methylene chloride or treated with acid chloride in the presence of triethylamine in methylene chloride. Then the resulting amides were treated with LiAlH_4_ in tetrahydrofuran (THF) to afford fendiline and its analogues. The structures of fendiline and its analogues (**2**–**17**) were identified by ^1^H-NMR spectra. As an example, the detailed synthetic procedure for the preparation of fendiline analogue **8** is provided as follows.

1-(2-Methylphenyl)ethanone (0.50 mL, 3.73 mmol) and ammonium acetate (2.88 g, 37.3 mmol) were dissolved in methanol (20 mL). To the stirred solution was added slowly a solution of NaCNBH_3_ (0.79 g, 11.2 mmol) dissolved in methanol (15 mL) through a dropping funnel. The whole mixture was stirred at room temperature. After stirring for 12 h, solvent was removed by using rotary evaporator. The residue was dissolved in ethyl acetate and then the solution was treated with 6 N HCl solution to make the solution to be acidic (pH = 12–13). The two layers were separated and then 6 N NaOH solution was added to the separated aqueous solution to make the solution to be basic (pH = 11–12). The basic aqueous solution was extracted with ethyl acetate twice and then combined organic solution was dried over anhydrous Na_2_SO_4_. Solvent was removed by using rotary evaporator to afford an intermediate amine B, 1-(2-methylphenyl)ethylamine (Ar = 2-methylphenyl, R_1_ = CH_3_, compound B in Figure 5) (0.21 g, 46% yield).

3,3-Diphenylpropionic acid (1.43 g, 10.5 mmol) was dissolved in methylene chloride (50 mL). To the solution was added EDC.HCl (2.22 g, 11.6 mmol) and DIEA (1.09 mL, 11.6 mmol). The mixture was stirred for 30 min at room temperature under an argon atmosphere. To the stirred solution was added 1-(2-methylphenyl)ethylamine (1.56 g, 16.6 mmol). After stirring for 12 h at room temperature, the whole mixture solution was treated with water (50 mL). The separated organic solution was dried over anhydrous Na_2_SO_4_. Solvent was removed by using rotary evaporator. The residue was purified by silica gel column chromatography (hexane/ethyl acetate, 95/5, v/v) to afford an intermediate amide C, 3,3-diphenyl-*N*-(1-(*o*-tolyl)ethyl)propanamide (Ar = 2-methylphenyl, R_1_ = CH_3_, R_2_ = 2,2-diphenylethyl, compound C in Figure 5) (1.0 g, 27.8% yield).

LiAlH_4_ (0.40 g) was dissolved in THF (20 mL). To the solution was added a solution of 3,3-diphenyl-*N*-(1-(*o*-tolyl)ethyl)propanamide (0.45 g, 1.31 mmol) dissolved in THF (10 mL) through a dropping funnel with stirring at 0 °C. And then the whole mixture was heated to reflux for 16 h. After removing solvent by using a rotary evaporator, the residue was dissolved in ethyl acetate. The ethyl acetate solution was washed with water. The organic solution was dried over anhydrous Na_2_SO_4_. Solvent was removed by using rotary evaporator. The residue was purified by silica gel column chromatography to afford fendiline analogue 8 (0.11 g, 40% yield). ^1^H-NMR (CDCl_3_, 300 MHz) δ 7.37–7.11 (m, 14H), 4.03–3.94 (m, 2H), 2.52–2.41 (m, 2H), 2.29–2.20 (m, 5H), 1.26 (d, 3H).

## 4. Conclusions

Fendiline and its sixteen analogues were resolved on CSP **1**. The separation factor (α) and resolution (R_S_) for fendiline were found to be 1.25 and 1.55, respectively, with the use of a mobile phase consisting of methanol–acetonitrile–trifluoroacetic acid–triethylamine at a ratio of 80/20/0.1/0.5 (v/v/v/v). In order to elucidate the structural characteristics required for the resolution of fendiline on CSP **1**, the chromatographic resolution behaviors for fendiline and its analogues were compared. The 3,3-diphenylpropyl group of fendiline and the difference in the steric bulkiness between the phenyl group and the methyl group at the chiral center of fendiline were found to be important for the chiral recognition. However, the exact chiral recognition mechanism needs further study.
